# Outcomes associated with under-dosing of rivaroxaban for management of non-valvular atrial fibrillation in real-world Japanese clinical settings

**DOI:** 10.1007/s11239-019-01934-6

**Published:** 2019-08-20

**Authors:** Takanori Ikeda, Satoshi Ogawa, Takanari Kitazono, Jyoji Nakagawara, Kazuo Minematsu, Susumu Miyamoto, Yuji Murakawa, Sanghun Iwashiro, Yoko Kidani, Yutaka Okayama, Toshiyuki Sunaya, Shoichiro Sato, Satoshi Yamanaka

**Affiliations:** 1 Department of Cardiovascular Medicine, Toho University Graduate School of Medicine, 6-11-1 Omorinishi, Ota-ku, Tokyo, 143-8541 Japan; 2 International University of Health & Welfare Mita Hospital, Tokyo, Japan; 3 Department of Medicine and Clinical Science, Graduate School of Medical Sciences, Kyushu University, Fukuoka, Japan; 4 Osaka Namba Clinic, Osaka, Japan; 5 National Cerebral and Cardiovascular Center, Suita, Osaka Japan; 6 Iseikai Medical Corporation, Osaka, Japan; 7 Department of Neurosurgery, Kyoto University Graduate School of Medicine, Kyoto, Japan; 8 The 4th Department of Internal Medicine, Teikyo University School of Medicine, Mizonokuchi Hospital, Kawasaki, Japan; 9 Medical Affairs Thrombosis, Medical Affairs, Bayer Yakuhin, Ltd., Osaka, Japan; 10 Pharmacovigilance & Medical Governance, Medical Affairs, Bayer Yakuhin, Ltd., Osaka, Japan; 11 Statistics & Data Insights, Data Sciences & Analytics, Research & Development Japan, Bayer Yakuhin, Ltd., Osaka, Japan

**Keywords:** Rivaroxaban, Anticoagulants, Underdosing, Atrial fibrillation, Stroke prevention

## Abstract

The approved dose of oral anticoagulant rivaroxaban for patients with non-valvular atrial fibrillation (NVAF) in Japan is 15 mg once daily (od) in patients whose creatinine clearance is ≥ 50 mL/min, but recent real-world studies have demonstrated that these patients often received less than the recommended dose due to bleeding concerns. The effect of under-dosing on safety and effectiveness outcomes remains unclear. We used 1-year follow-up data from the XAPASS, a real-world Japanese prospective, single-arm, observational study. Of the 11,308 patients, 6521 patients who completed a 1-year follow-up and had a creatinine clearance ≥ 50 mL/min were included in this sub-analysis. Primary endpoints were any bleeding and a composite of stroke/non-central nervous system systemic embolism (non-CNS SE)/myocardial infarction (MI). Among the 6521 patients, 4185 (64.2%; mean CHADS_2_ score: 1.8) received the 15 mg od (recommended dose), whereas 2336 (35.8%; mean CHADS_2_ score: 2.3) received 10 mg od (under-dose). After adjusting for patient characteristics by propensity scoring and inverse probability of treatment weighting, incidence rates of major bleeding were comparable between under-dosed patients and patients who received the recommended dose (1.34 vs. 1.63 events/100 patient-years, *p* = 0.197), although the incidence rates of stroke/non-CNS SE/MI were higher in under-dosed patients than in those who received the recommended dose (2.15 vs. 1.48 events/100 patient-years, *p* = 0.009). In Japanese clinical practice, some NVAF patients receive rivaroxaban doses inconsistent with the recommendation. Considering the total clinical benefit, the recommended dose may be preferable in terms of balance of safety and effectiveness.

*Clinicaltrials.gov* NCT01582737.

**Electronic supplementary material:**

The online version of this article (10.1007/s11239-019-01934-6) contains supplementary material, which is available to authorized users.

## Highlights

- Japanese patients with non-valvular atrial fibrillation (NVAF) who were at a higher risk for stroke and bleeding were more likely to be prescribed under-dose of rivaroxaban than those who were at a lower risk.
- After adjustment for patient background, under-dosing of rivaroxaban in Japanese patients with NVAF and creatinine clearance ≥ 50 mL/min was associated with a decreased incidence of bleeding and an increased incidence of stroke/non-central nervous system systemic embolism (non-CNS SE)/myocardial infarction (MI).
- The most common reasons that physicians prescribed an under-dose of rivaroxaban were high bleeding risk, followed by elderly age and renal impairment.
- Considering the total clinical benefit of NVAF patients, the recommended dose may be preferable in terms of the balance of safety and effectiveness.

## Introduction

Atrial fibrillation (AF), one of the most common cardiac arrhythmias in Japan, affects 0.6% of Japanese [1]. AF increases the risk of ischemic stroke by fivefold [2]. To reduce the risk of ischemic stroke, direct oral anticoagulants (DOACs) such as rivaroxaban are widely prescribed to patients with AF.

Rivaroxaban is a direct factor Xa inhibitor. Its safety and effectiveness in patients with non-valvular AF (NVAF) were examined in two phase III clinical trials, the worldwide ROCKET AF study [3] and the J-ROCKET AF study [4], which focused on Japanese patients and Japan-specific rivaroxaban dosages. In these studies, rivaroxaban was demonstrated to be non-inferior to warfarin for the prevention of stroke and systemic embolism in ROCKET AF study, and for the principal safety outcome of major and non-major clinical relevant bleeding in J–ROCKET AF study.

In these two clinical trials, patients were prescribed rivaroxaban strictly according to the dosage recommendations. In contrast, in real-world clinical practice, physicians might prescribe anticoagulants at doses inconsistent with recommendations due to, for example, a high bleeding risk in specific patients. In fact, under-dosing of DOACs has been reported in several real-world studies in Japan, including the EXPAND study [5], the SAKURA AF Registry [6, 7], and the KiCS-AF registry [8]. Under-dosing of DOACs is primary concern in daily clinical practice. Although several investigations have been reported on under-dosing of DOACs [9-11], the effect of under-dosing on clinical outcomes remains controversial.

The XAPASS, a real-world, prospective, single-arm, observational study, was aimed at examining the safety and effectiveness of rivaroxaban in daily Japanese clinical practice [12]. In the XAPASS, 35.8% of patients with NVAF who had a creatinine clearance (CrCl) ≥ 50 mL/min received an inappropriate under-dose of rivaroxaban [13]. In the current sub-analysis of the XAPASS, we analyzed the 1-year safety and effectiveness outcomes of patients who received an under-dose compared with those who received the recommended dose.

## Methods

The design of the XAPASS was described previously [12]. Briefly, the XAPASS was a real-world, prospective, single-arm, observational study approved by the Ministry of Health, Labour, and Welfare in Japan. It was performed in accordance with the Good Post-marketing Study Practice standards of this Ministry. The current sub-analysis of the XAPASS investigated the 1-year safety and effectiveness outcomes in patients with CrCl ≥ 50 mL/min, comparing those who received the recommended dose of rivaroxaban [15 mg once daily (od)] with those who were under-dosed (10 mg od).

### Patients and treatment

The XAPASS included 11,308 patients with NVAF who were beginning rivaroxaban treatment to reduce their risks of ischemic stroke and systemic embolism (SE). In the XAPASS, 9578 patients had completed follow-up for at least 11 months, discontinued rivaroxaban treatment within 1 year, or were lost to follow-up within 1 year [13]. Of these patients, the 6521 who had CrCl ≥ 50 mL/min were included in this sub-analysis. Patients were treated with oral rivaroxaban at a dose of either 15 mg od or 10 mg od which are approved doses in Japan. The dosage and treatment duration were decided by the treating physicians. If physicians prescribed under-dose rivaroxaban, they were asked to write the reasons for under-dosing on surveillance sheets.

### Safety and effectiveness outcomes

The primary safety outcome was any bleeding, including both major and non-major bleeding. Major bleeding was defined as the International Society of Thrombosis and Haemostasis (ISTH) criteria; non-major bleeding was defined as any overt bleeding not meeting those criteria.

The primary effectiveness outcome was a composite of stroke (hemorrhagic or ischemic), non-central nervous system (non-CNS) SE, and myocardial infarction (MI), all defined previously [12].

### Statistical analysis

Patient characteristics were compared between patients who received recommended dose rivaroxaban and under-dose rivaroxaban. To adjust for patient characteristics and enable direct comparison of outcomes between the two groups, propensity scoring [14] and inverse probability of treatment weighting [15] were performed. Propensity scores were estimated using logistic regression based on baseline characteristics [age, sex, body weight, and comorbidities, including congestive heart failure, hypertension, diabetes mellitus, prior stroke/transient ischemic attack (TIA), and vascular disease]. The standardized difference was calculated to compare the patient characteristics between the two groups [16].

A log-rank test was conducted for overall survival curves. Hazard ratios were based on COX proportional hazards models. Predictive factors for prescription of under-dose were investigated by stepwise selection using the Cox proportional hazards model with a significance level of 5%. Explanatory variables of medical interest were selected considering both data availability and multi-collinearity. The statistical analyses were performed using SAS software, version 9.2 or higher (SAS Institute, Inc., Cary, NC, USA).

## Results

### Patients

Patient characteristics are shown in Table 1. Of the 6521 patients in this study, 4185 (64.2%; mean CHADS_2_ score: 1.8) received the recommended rivaroxaban dose of 15 mg od, and 2336 (35.8%; mean CHADS_2_ score: 2.3) received under-dose rivaroxaban (10 mg od). Two patients in recommended dose group were excluded during adjustment due to missing data. Patients who received under-dose rivaroxaban were more likely to be ≥ 75 years old, female, and ≤ 50 kg, and less likely to have CrCl ≥ 80 mL/min than the patients who received the recommended dose. Furthermore, patients who received under-dose rivaroxaban had higher CHADS_2_, CHA_2_DS_2_-VASc, and modified HAS-BLED scores and higher rates of baseline comorbidities, including congestive heart failure, hypertension, prior ischemic stroke/TIA, and vascular disease, and were more likely to use antiplatelets. After adjustment, these characteristics were similar between the two groups, as demonstrated by the standardized difference being less than 0.1 in most of the categories [16].

**Table 1:** Patient characteristics

Characteristic	Before adjustment			After adjustment		
	Recommended dose (15 mg)	Under-dose (10 mg)	Standardized difference	Recommended dose (15 mg)	Under-dose (10 mg)	Standardized difference
Number of patients	4185	2336		4183	2336	
Age (years)	68.0 ± 9.0	74.8 ± 7.7	0.811	70.3 ± 11.1	68.0 ± 21.2	0.134
≥ 75	23.6	58.1	0.750	33.6	35.1	0.030
Female sex	27.1	40.0	0.277	31.6	29.3	0.050
Body weight (kg)	66.26 ± 12.31	62.40 ± 11.85	0.319	64.95 ± 15.05	65.85 ± 22.59	0.047
≤ 50	7.6	16.5	0.276	9.4	12.4	0.096
BMI (kg/m^2^)	24.73 ± 3.93	24.57 ± 4.09	0.040	24.62 ± 4.90	25.03 ± 7.18	0.067
SCr (mg/dL)	0.801 ± 0.178	0.797 ± 0.207	0.020	0.786 ± 0.223	0.841 ± 0.382	0.174
CrCl (mL/min)	82.6 ± 30.5	69.0 ± 18.6	0.538	79.2 ± 36.4	78.2 ± 45.0	0.025
50 to < 80	53.5	78.2	0.539	60.0	62.7	0.056
≥ 80	46.5	21.8	0.539	40.0	37.7	0.056
CHADS_2_ score						
Mean score	1.8 ± 1.2	2.3 ± 1.3	0.375	1.9 ± 1.6	2.0 ± 2.3	0.004
0	13.1	6.3	0.232	11.1	12.0	0.027
1	32.4	22.9	0.213	29.4	28.4	0.022
2	28.5	32.3	0.082	29.7	28.7	0.023
3	15.7	20.3	0.118	17.2	18.4	0.033
4	8.0	12.6	0.153	9.3	8.7	0.022
5	1.9	4.7	0.155	2.7	3.3	0.036
6	0.4	1.0	0.070	0.7	0.6	0.012
CHA_2_DS_2_-VASc score						
Mean score	2.8 ± 1.5	3.6 ± 1.5	0.554	3.1 ± 1.9	3.0 ± 2.8	0.043
0	4.6	1.0	0.216	3.3	5.6	0.115
1	15.6	6.2	0.306	12.2	13.7	0.045
2	25.0	14.2	0.274	22.2	19.3	0.071
3	23.9	27.9	0.091	24.3	26.6	0.053
4	17.6	24.2	0.163	20.6	17.5	0.078
5	9.1	15.0	0.180	11.2	10.3	0.030
6	3.5	8.7	0.220	4.7	5.4	0.028
7	0.7	2.4	0.139	1.3	1.3	0.001
8	0.1	0.4	0.065	0.2	0.2	0.009
9	0.02	0.04	0.010	0.05	0.02	0.015
Modified HAS-BLED score^a^						
Mean score	1.2 ± 0.9	1.6 ± 0.9	0.504	1.3 ± 1.1	1.3 ± 1.7	0.061
0	22.2	6.6	0.455	16.7	19.9	0.082
1	47.3	43.9	0.068	49.5	39.7	0.199
2	23.4	34.6	0.247	25.8	28.9	0.069
3	6.0	11.7	0.202	6.7	8.8	0.077
4	0.9	3	0.151	1.1	2.6	0.116
5	0.1	0.1	0.010	0.1	0.1	0.005
6	0	0.04	0.029	0	0.02	0.019
7	0	0		0	0	
8	0	0		0	0	
Baseline comorbidities						
Congestive heart failure	19.4	23.9	0.110	20.8	20.6	0.005
Hypertension	73.2	76.9	0.086	74.4	73.2	0.028
Diabetes mellitus	23.5	23.1	0.009	23.4	23.5	0.003
Prior ischemic stroke/TIA	19.6	22.0	0.059	20.6	20.7	0.004
Vascular disease^b^	2.6	4.2	0.088	3.2	3.6	0.020
Type of AF						
Paroxysmal	34.9	35.4	0.009	34.3	37.3	0.063
Persistent	36.0	35.6	0.002	36.4	34.8	0.032
Permanent	23.8	23.6	0.005	23.4	22.6	0.021
Other	0.3	0.3	0.001	0.3	0.5	0.026
Unknown	5.3	5.2	0.004	5.6	4.8	0.035
Oral antiplatelet use	12.8	15.3	0.071	13.5	13.1	0.011

Data are presented as % or mean ± standard deviation

*BMI* body mass index, *SCr* serum creatinine, *CrCl* creatinine clearance, *TIA* transient ischaemic attack, *AF* atrial fibrillation

^a^Maximum score is 8 because of the exclusion of the factor "labile INR" from the HAS-BLED score. *INR* international normalized ratio

^b^Vascular disease is defined as myocardial infarction and/or peripheral artery disease and or aortic plaque

### Treatment

The mean treatment duration was 305.1 ± 116.1 days for patients who received the recommended dose rivaroxaban and 308.6 ± 111.7 days for those who received under-dose rivaroxaban. Of the 4183 patients who received the recommended dose, 2917 (69.7%) continued rivaroxaban treatment; 723 (17.3%) were lost to follow-up, including patient transfer; and 545 (13.0%) discontinued rivaroxaban treatment. Of the 2336 patients who received the under-dose, 1579 (67.6%) continued rivaroxaban treatment; 458 (19.6%) were lost to follow-up, including patient transfer; and 299 (12.8%) discontinued rivaroxaban treatment.

### Safety and effectiveness outcomes

**Fig. 1:**
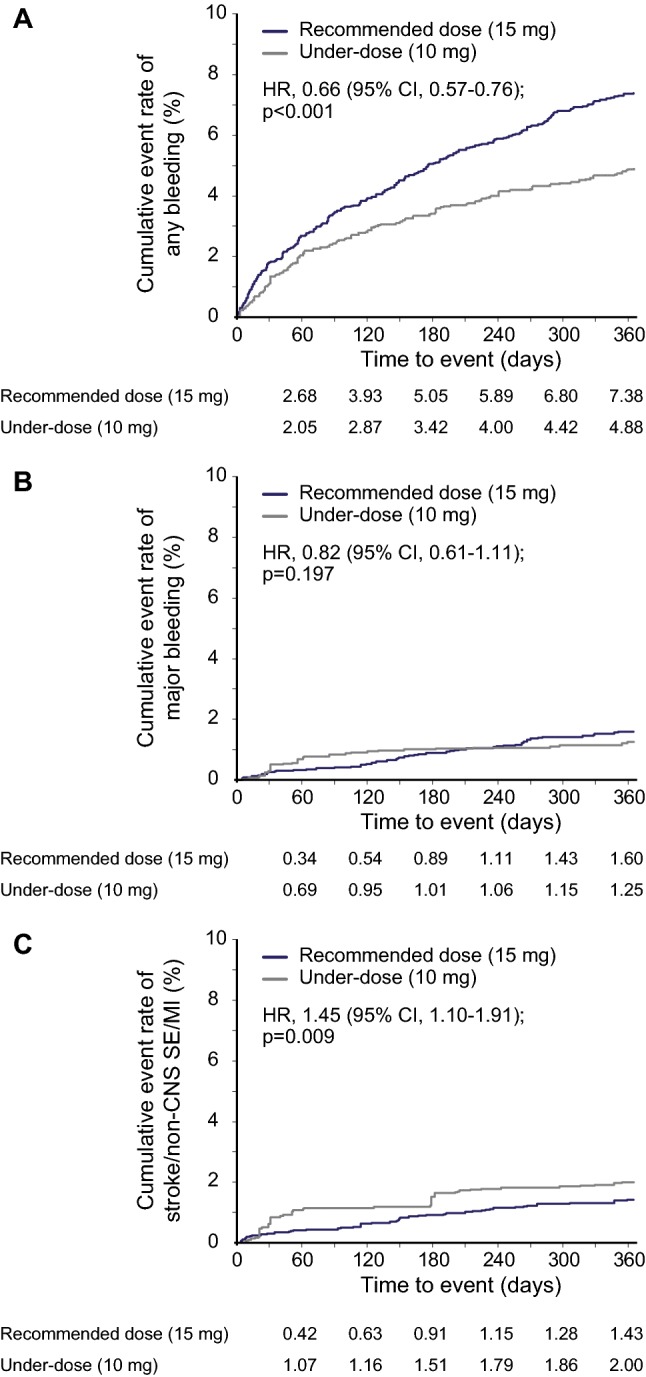
Cumulative rates of **a** any bleeding, **b** major bleeding, and **c** stroke/non-CNS SE/MI in patients who received the recommended dose of rivaroxaban [15 mg once daily (od)] versus patients who received under-dose rivaroxaban (10 mg od). *CI* confidence interval, *HR* hazard ratio, *non-CNS SE* non-central nervous system systemic embolism, *MI* myocardial infarction

After adjustment, under-dose of rivaroxaban resulted in a lower incidence rate of the primary safety outcome, any bleeding [5.29 vs. 8.05 events/100 patient-years, hazard ratio (HR) 0.66, 95% confidential interval (CI) 0.57–0.76; Table 2 and Fig. 1a]. There was no significant difference in major bleeding between patients who received the under-dose and those who received the recommended dose (1.34 vs. 1.63 events/100 patient-years, HR 0.82, 95% CI 0.61–1.11; Table 2 and Fig. 1b). Among components of ISTH definition of major bleeding, the incidence rate of hemoglobin decrease ≥ 2 g/dL was lower in the under-dose group (0.34 vs. 0.63 events/100 patient-years, HR 0.55, 95% CI 0.32–0.95; Table 2).

**Table 2:** Safety and effectiveness outcomes after adjusting for baseline patient characteristics

Safety outcome	Incidence rate, events/100 patient-years (95% CI)		HR (95% CI), Under-dose group versus recommended dose group	*p* value
	Recommended dose (N = 4183)	Under-dose (N = 2336)		
Any bleeding	8.05 (7.29–8.80)	5.29 (4.70–5.87)	0.66 (0.57–0.76)	< 0.001
Major bleeding	1.63 (1.30–1.97)	1.34 (1.05–1.63)	0.82 (0.61–1.11)	0.197
Fatal bleeding	0.14 (0.04–0.24)	0.06 (0.00–0.13)	0.44 (0.13–1.49)	0.186
Critical organ bleeding	0.75 (0.52–0.97)	0.82 (0.59–1.05)	1.09 (0.72–1.65)	0.671
Intracranial hemorrhage	0.64 (0.43–0.85)	0.75 (0.54–0.97)	1.18 (0.76–1.83)	0.455
Hemoglobin decrease ≥ 2 g/dL	0.63 (0.42–0.83)	0.34 (0.20–0.49)	0.55 (0.32–0.95)	0.031
Transfusion of ≥ 2 units of packed RBC or whole blood	0.15 (0.05–0.25)	0.08 (0.01–0.16)	0.58 (0.19–1.75)	0.334

Effectiveness outcome	(N = 4168)		(N = 2326)	
Stroke/non-CNS SE/MI	1.48 (1.16–1.80)	2.15 (1.78–2.52)	1.45 (1.10–1.91)	0.009
Ischemic stroke	1.09 (0.81–1.36)	1.26 (0.97–1.54)	1.15 (0.49–1.47)	0.414
Hemorrhagic stroke	0.53 (0.34–0.72)	0.52 (0.34–0.71)	0.99 (0.60–1.63)	0.957
non-CNS SE	0.04 (0.00–0.09)	0.27 (0.14–0.40)	7.59 (1.76–32.7)	0.007
Myocardial infarction	0.05 (0.00–0.11)	0.09 (0.01–0.16)	1.78 (0.41–7.76)	0.443
Ischemic stroke/non-CNS SE/MI	1.14 (0.86–1.42)	1.62 (1.30–1.94)	1.42 (1.03–1.95)	0.030

Baseline characteristics were adjusted by propensity scoring and inverse probability of treatment weighting

*p* values were calculated by Wald tests, p-value less than 5% was considered nominally statistically significant

*CI* confidence interval, *HR* hazard ratio, *RBC* red blood cells, *CNS* central nervous system, *SE* systemic embolism, *MI* myocardial infarction

Under-dose of rivaroxaban resulted in a higher incidence rate of the primary effectiveness outcome, stroke/non-CNS SE/MI (2.15 vs. 1.48 events/100 patient-years, HR 1.45, 95% CI 1.10–1.91; Table 2 and Fig. 1c). It also resulted in higher incidence rates of non-CNS SE as an individual endpoint (0.27 vs. 0.04 events/100 patient-years, HR 7.59, 95% CI1.76–32.7) and ischemic stroke/non-CNS SE/MI (1.62 vs. 1.14 events/100 patient-years, HR 1.42, 95% CI 1.03–1.95; Table 2).

**Fig. 2:**
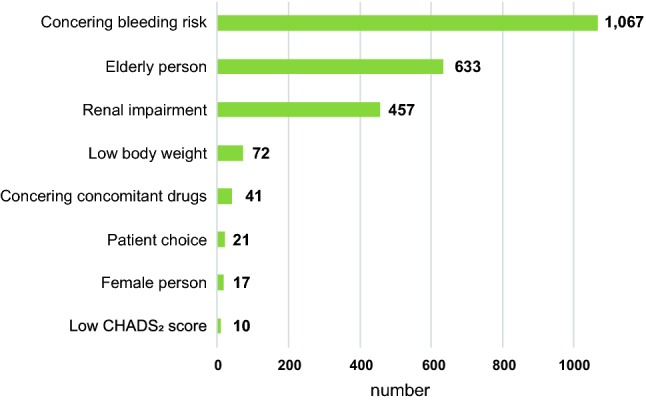
The reasons for prescribing under-dose rivaroxaban from surveillance sheets (overlapping exists)

The reasons for prescribing under-dose rivaroxaban, as recorded on surveillance sheets, are listed in Fig. 2. The top reasons were high bleeding risk, followed by elderly age and renal impairment. These data are supported by the results of multivariable analysis and stepwise methods, which demonstrated elderly age, female sex, low body weight, high serum creatinine level, or comorbidity of congestive heart failure or vascular disease as factors associated with the under-dose description (Table S1).

## Discussion

This sub-analysis of the XAPASS investigated the effect of rivaroxaban under-dosing on clinical outcomes in patients with NVAF. The incidence rate of the primary safety endpoint, any bleeding, was lower in patients who received the under-dose rivaroxaban, whereas that of major bleeding was comparable between the two groups. Moreover, the incidence rate of the primary effectiveness endpoint, stroke/non-CNS SE/MI, was higher in the under-dose group.

According to surveillance sheets, the primary reasons for prescribing under-dose rivaroxaban were bleeding risk, followed by elderly age and renal impairment (Fig. 2). Similarly, according to multivariable analysis and stepwise methods, physicians tended to prescribe under-dose rivaroxaban to patients who were elderly, female sex, low body weight or who had high creatinine levels, congestive heart failure, or vascular disease (Table S1). These analyses might reflect the prescription intentions of treating physicians. Among baseline comorbidities, only congestive heart failure and vascular disease were predictors of under-dosing.

In the present study, the percentage of patients with CrCl ≥ 50 mL/min who received under-dose rivaroxaban was 35.8%. This percentage was comparable to that of the EXPAND study, another real-world Japanese study that focused on the safety and effectiveness of rivaroxaban for stroke prevention in patients with NVAF, where 1609 (30.2%) of the 5326 patients with CrCl ≥ 50 mL/min received under-dose rivaroxaban [5]. The slight difference may have been due to differences in the study populations or the prescribing behavior of physicians at the various clinical sites.

After adjustment for baseline characteristics in the current study, the incidence rates of major bleeding were similar in patients who were under-dosed and in those who received the recommended dose of rivaroxaban (1.34 vs. 1.63 events/100 patient-years, HR 0.82, 95% CI 0.61–1.11). Similarly, the incidence rates of major bleeding in the EXPAND study were comparable in patients who received under-dose rivaroxaban and those who received the recommended dose (1.1% vs. 1.0% per year, *p* = 0.506) [5]. In the current study, the post-adjustment incidence rate of stroke/non-CNS SE/MI was significantly higher in patients who received under-dose rivaroxaban compared with those who received the recommended dose (2.15 vs. 1.48 events/100 patient-years, *p* = 0.009), whereas in the EXPAND study, the incidence rates of stroke/SE were also comparable between the two patient groups (0.9% vs. 0.8%/year, *p* = 0.795) [5]. While both the safety and effectiveness results of the current study appear to conflict with those of the EXPAND study, the two studies cannot be directly compared since, unlike our study results, the EXPAND study results were not adjusted for patient characteristics.

The SAKURA AF Registry and the KiCS-AF registry are both multicenter registries in Japan which included NVAF patients prescribed any oral anticoagulants (OACs) [6-8, 17]. In the SAKURA AF registry, patients who received under-dose DOACs had a high risk background compared to those who received the recommended dose and had a lower risk background than those who received appropriate reduced-dose, which was the tendency shown in the KiCS-AF registry [7, 8]. In the SAKURA AF registry, incidence rates of stroke/SE and major bleeding were both comparable between patients who received under-dose DOACs and those who received recommended dose DOACs [7].

The XANTUS study was a worldwide, prospective, observational study that also reported outcomes associated with non-recommended dosing of rivaroxaban, including both over-dosing and under-dosing [18]. In the XANTUS study, of the 3,794 patients with a CrCl ≥ 50 mL/min, only 583 (15.4%) patients received under-dose rivaroxaban (15 mg od in a global population). Compared with patients who received the recommended dose (20 mg od), the patients who received under-dose rivaroxaban were higher-risk patients, more likely to be > 75 years old, less likely to have CrCl > 80 mL/min, and more likely to have comorbidities (hypertension, diabetes mellitus, prior stroke/SE/TIA, and congestive heart failure) and higher CHADS_2_, CHA_2_DS_2_-VASc, and HAS-BLED scores [19]. The trends in these studies are similar to the trends in our analysis.

In the XANTUS sub-analysis focused on outcomes associated with non-recommended dosing of rivaroxaban, the incidence rates in patients who received under-dosed rivaroxaban showed 3.9 events/100 patient-years for major bleeding and 2.7 events/100 patient-years for thromboembolic events (stroke, TIA, non-CNS SE, or MI) [19]. The rates in patients who received the recommended dose (20 mg od for patients with CrCl ≥ 50 mL/min and 15 mg od for patients with CrCl < 50 mL/min) were 2.6 events/100 patient-years and 1.9 events/100 patient-years, respectively. Although these incidence rates were higher in patients who received under-dose rivaroxaban compared to patients who received the recommended dose, statistical analysis has not been performed to date. The authors reported that the incidence rate of composite of major bleeding, stroke/non-CNS SE, or all-cause death was comparable between the two groups after adjustment for patients’ characteristics (HR for under-dosed vs. recommended dose 1.10, 95% CI 0.77–1.58).

## Limitations

The current sub-analysis had several limitations. The follow-up period was limited, and the incidence rates might have been affected by treatment duration. Also, the loss of patients to follow-up might have led to an underestimation of event rates. Moreover, this sub-analysis was based only on the initial rivaroxaban dose and baseline patient characteristics: it did not account for changes in dose or CrCl during the study, or for invasive treatment, such as catheter ablation or surgery. Another limitation is that, to avoid a reduction in patient numbers when adjusting for patient characteristics, variables of the propensity score were selected based on data availability and clinical importance. Because of this limitation, some clinical characteristics and details of antiplatelet agents were not completely matched between the two dose cohorts, which may have affected the results.

## Conclusions

The 1-year data of the XAPASS showed that patients at a higher risk of stroke and bleeding were more likely to be prescribed under-dose rivaroxaban compared to patients at lower risk. Under-dosing of rivaroxaban was associated with a decreased incidence of any bleeding and an increased incidence of stroke/non-CNS SE/MI. Considering the total clinical benefit of NVAF patients, the recommended dose may be preferable in terms of the balance of safety and effectiveness in Japanese real-world settings.

## Electronic supplementary material

Below is the link to the electronic supplementary material.

Supplementary file1 (DOCX 44 kb)
