# Supplementary material for: Outcomes associated with under-dosing of rivaroxaban for management of non-valvular atrial fibrillation in real-world Japanese clinical settings
Source: J Thromb Thrombolysis. 2019 Aug 20;48(4):653–60. doi: 10.1007/s11239-019-01934-6 (PMC6800859; doi:10.1007/s11239-019-01934-6)
Supplement: Supplementary file 1 — Supplementary file1 (DOCX 44 kb) [file 11239_2019_1934_MOESM1_ESM.docx]

**Supplementary**

**Table S1. Logistic regression analysis of trends in prescribing rivaroxaban under-dose**

|  | | **Univariable analysis** | |  | **Multivariable analysis** | |  | **Stepwise analysis** | |
| --- | --- | --- | --- | --- | --- | --- | --- | --- | --- |
| **Variables*** | | **Odds ratio (95% CI)** | ***P* value** |  | **Odds ratio (95% CI)** | ***P* value** |  | **Odds ratio (95% CI)** | ***P* value** |
| **Age, years** | |  |  |  |  |  |  |  |  |
|  | ≥75 vs. <75 | 4.48 (4.02–5.00) | <0.001 |  | 4.36 (3.90–4.88) | <0.001 |  | 4.36 (3.90–4.87) | <0.001 |
| **Sex** | |  |  |  |  |  |  |  |  |
|  | Female vs. Male | 1.80 (1.62–2.00) | <0.001 |  | 1.54 (1.35–1.74) | <0.001 |  | 1.54 (1.36–1.74) | <0.001 |
| **Body weight, kg** | |  |  |  |  |  |  |  |  |
|  | ≤50 vs. >50 | 2.41 (2.05–2.82) | <0.001 |  | 2.03 (1.68–2.44) | <0.001 |  | 1.99 (1.66–2.39) | <0.001 |
| **SCr** | |  |  |  |  |  |  |  |  |
|  | ≥0.9 mg/dL (males), or ≥0.75 mg/dL (females) | 1.20 (1.08–1.34) | <0.001 |  | 1.65 (1.46–1.86) | <0.001 |  | 1.65 (1.47–1.86) | <0.001 |
| **Baseline comorbidities** | |  |  |  |  |  |  |  |  |
|  | Congestive heart failure | 1.31 (1.16–1.48) | <0.001 |  | 1.22 (1.07–1.39) | 0.003 |  | 1.24 (1.08–1.41) | 0.002 |
|  | Hypertension | 1.22 (1.08–1.37) | <0.001 |  | 1.07 (0.94–1.22) | 0.309 |  |  |  |
|  | Diabetes mellitus | 0.98 (0.87–1.10) | 0.734 |  | 1.05 (0.92–1.17) | 0.476 |  |  |  |
|  | Prior stroke/TIA | 1.16 (1.02–1.31) | 0.022 |  | 0.96 (0.84–1.10) | 0.584 |  |  |  |
|  | Vascular disease^#^ | 1.64 (1.24–2.16) | <0.001 |  | 1.65 (1.21–2.23) | 0.001 |  | 1.69 (1.25–2.27) | <0.001 |
|  | Hepatic dysfunction | 0.95 (0.78–1.17) | 0.643 |  | 1.07 (0.86–1.34) | 0.547 |  |  |  |
| **Oral antiplatelet use** | | 1.08 (0.85–1.39) | 0.523 |  | 1.04 (0.79–1.36) | 0.797 |  |  |  |
| *Variables were selected from baseline data  Data availability was 99.9%. Stepwise regression analysis was performed with a significance level of 5%. | | | | | | | | | |
| SCr, serum creatinine; TIA, transient ischemic attack | | | | | | | | | |
| ^#^Vascular disease was defined as myocardial infarction, peripheral artery disease, and/or aortic plaque. | | | | | | | | | |
